# Editorial: New discoveries in the field of brain stimulation and addiction disorders

**DOI:** 10.3389/fnins.2023.1337773

**Published:** 2023-11-29

**Authors:** Dongyu Kang, Travis E. Baker, Vaughn R. Steele

**Affiliations:** ^1^Department of Psychiatry, School of Medicine, Yale University, New Haven, CT, United States; ^2^Center for Molecular and Behavioral Neuroscience, Rutgers University—Newark, Newark, NJ, United States; ^3^Olin Neuropsychiatry Research Center, Institute of Living, Hartford, CT, United States

**Keywords:** non-invasive brain stimulation (NIBS), repetitive transcranial magnetic stimulation, deep brain stimulation, functional magnetic resonance imaging (fMRI), electroencephalography

Substance use disorders (SUDs) are a chronic, relapsing brain disease of dysregulated circuits (Volkow et al., 2016). These dysregulated circuits are malleable and targetable with repetitive transcranial magnetic stimulation (rTMS), a type of non-invasive brain stimulation (Diana et al., 2017; Ekhtiari et al., 2019; Steele, 2020a; Steele and Maxwell, 2021; NIBS). Targeting a small cortical seed with rTMS stimulation affects the neural tissue at the target location and propagates downstream to secondary regions (Diana et al., 2017; Siebner et al., 2022) allowing a neuromodulation window into sub-cortical brain regions. Invasive brain stimulation techniques, such as deep brain stimulation (DBS) also hold promise in this area of psychiatric disorders (Williams and Okun, 2013). Considering many clinical populations have dysregulated circuits that are difficult to directly stimulate (Goodkind et al., 2015; McTeague et al., 2017), rTMS holds tremendous potential to treat myriad diseases (Rosson et al., 2022). The field is aware of many parameters to interrogate to optimized application of stimulation to treat SUDs (Steele and Maxwell, 2021). In particular, quantifying dysregulated neural circuits and proximal functions using well-established neuropsychological and neuroimaging measures [e.g., electroencephalography, functional magnetic resonance imaging (fMRI)] can define new targets for NIBS, tailor NIBS protocols for specific circuits and dysfunction, and more objectively measure the efficacy and outcome of NIBS (See for further discussion: Ekhtiari et al., 2019; Steele, 2020a,b; Steele and Maxwell, 2021). Such advances will add to our understanding of the cognitive dysfunctions underlying SUDs and the therapeutic mechanisms of action of NIBS. This Research Topic highlights recent work in this area toward developing stimulation applications to treat SUDs and other psychiatric disorders.

## Overview of the Research Topic

Wesley and Lile reported a promising research framework combining rTMS with behavioral pharmacology to study mechanisms of SUDs, both of which have been well-studied to be effective for treating psychiatric disorders. They highlight a method of capturing dose-response of THC and rTMS with behavioral and fMRI measures. Novel integration of interventions could be a key component to optimizing treatment protocols for SUDs.

Shaheen et al. conducted a meta-analysis to evaluate the effect of DBS for SUDs. DBS, primarily targeting the nucleus accumbens, has favorable effects with a 56% reduction in the clinical scores and 8% of relapse rate which is better than the alternative relapse rate of 85% without DBS. Although these effects are promising, heterogeneity across studies hinder definitive conclusions other than DBS has promise to treat SUDs.

Demographic factors have a significant impact on rTMS efficacy, as explored by Gersner et al.. In their secondary data analysis of smoking cessation, responders and non-responders to their rTMS intervention were differentiated by demographic variables such as age, education, race, and overall smoking history. This highlights more parameters researchers may consider in future trials.

Finally, a novel method of rTMS targeting is reported by Cao et al.. They identified a clinically relevant and rTMS targetable network derived from with resting-state fMRI. Validation experiments included interventions for schizophrenia and depression and produced promising results suggesting individualized rTMS targeting across psychiatric disorders is possible.

## Conclusion

The articles included in this Research Topic highlighted future directions for neuromodulation (rTMS and DBS) applications for SUDs and other psychiatric disorders. Preliminary applications of rTMS as a treatment for SUDs are promising (Steele et al., 2019; e.g., McCalley et al., 2023), there are many parameters yet to be fully explored (Steele and Maxwell, 2021).

We believe uncovering the neural mechanism of stimulation in SUD samples is the most important area to address in the future (Steele, 2021). Straightforward application of rTMS combined with measures of neural activation are possible to uncover how best to apply these methods as a treatment for SUDs (e.g., Biernacki et al., 2020). In this Research Topic, we highlight three papers seeking to better understand the neural underpinnings of stimulation to affect positive change in clinical populations (Cao et al.; Gersner et al.; Wesley and Lile). Combining rTMS with behavioral pharmacology methods presented by Wesley and Lile provided a framework to examine does-related effect of rTMS intervention and mechanisms of SUD. The meta-analysis on DBS presented robust evidence of the therapeutic effect provided by a circuit-based stimulation methods (Shaheen et al.). Ultimately, one of the biggest questions is knowing which circuit to target and how. Cao et al. made strides in this direction by using fMRI to develop individualized rTMS targets and then validated their methods in new participants. The studies included in this Research Topic address unique areas necessary for the field to understand as we develop neuromodulation parameters for interventions.

Together, this Research Topic highlights recent advances in the field but also how much more there is to accomplish. New findings are rapidly emerging in this exciting area of research. Optimizing neuromodulation as an effective therapeutic for SUDs and other clinical populations will take time and multiple groups working together to tackle the large issues in the field. Consortiums (e.g., Ekhtiari et al., 2019, 2020) and highlighting potential areas to address (Steele, 2020a, 2021) are helpful. As highlighted here, the field is collectively working to address these issues and move neuromodulation applications toward viable treatments for SUDs and other psychiatric disorders.

## Author contributions

DK: Conceptualization, Writing—original draft, Writing—review & editing. TB: Conceptualization, Writing—review & editing. VS: Conceptualization, Supervision, Writing—review & editing.
